# Diabetic kidney disease in type 2 diabetes: a review of pathogenic mechanisms, patient-related factors and therapeutic options

**DOI:** 10.7717/peerj.11070

**Published:** 2021-04-19

**Authors:** Louise Woodhams, Tin Fei Sim, Leanne Chalmers, Bu Yeap, Daniel Green, Markus Schlaich, Carl Schultz, Graham Hillis

**Affiliations:** 1 Curtin Medical School, Curtin University of Technology, Perth, Western Australia, Australia; 2 Department of Endocrinology and Diabetes, Fiona Stanley Hospital, Perth, Western Australia, Australia; 3 Medical School, The University of Western Australia, Perth, Western Australia, Australia; 4 School of Human Sciences (Exercise and Sport Sciences), The University of Western Australia, Perth, Western Australia, Australia; 5 Department of Cardiology and Nephrology, Royal Perth Hospital, Perth, Western Australia, Australia; 6 Neurovascular Hypertension and Kidney Disease Laboratory, Baker Heart and Diabetes Institute, Melbourne, Victoria, Australia; 7 Dobney Hypertension Centre, School of Medicine, Royal Perth Hospital Unit/Medical Research Foundation, The University of Western Australia, Perth, Western Australia, Australia; 8 Department of Cardiology, Royal Perth Hospital, Perth, Western Australia, Australia

**Keywords:** Type 2 Diabetes Mellitus, Diabetic kidney disease, Pathogenesis, Risk factors

## Abstract

The global prevalence of diabetic kidney disease is rapidly accelerating due to an increasing number of people living with type 2 diabetes. It has become a significant global problem, increasing human and financial pressures on already overburdened healthcare systems. Interest in diabetic kidney disease has increased over the last decade and progress has been made in determining the pathogenic mechanisms and patient-related factors involved in the development and pathogenesis of this disease. A greater understanding of these factors will catalyse the development of novel treatments and influence current practice. This review summarises the latest evidence for the factors involved in the development and progression of diabetic kidney disease, which will inform better management strategies targeting such factors to improve therapeutic outcomes in patients living with diabetes.

## Introduction

Rapid urbanisation and increasingly sedentary lifestyles have increased the global prevalence of type 2 diabetes mellitus (T2DM) over recent decades (Guariguata et al., 2014). According to the International Diabetes Federation, an estimated 451 million people are currently living with diabetes worldwide, with the number expected to increase to 693 million by 2045 (Cho et al., 2018). Poorly managed T2DM may lead to complications, negatively impacting on health outcomes. Diabetic kidney disease (DKD) is the most common cause of chronic and end-stage kidney disease (ESKD), with approximately one in three patients living with T2DM developing DKD (Gheith et al., 2016). The increasing prevalence of T2DM and the significant morbidity, mortality and healthcare costs associated with DKD (Gheith et al., 2016) have led to a research imperative to better understand the pathogenesis of DKD and develop better management strategies. The current treatment approach for preventing microvascular complications in T2DM, such as DKD, comprises of a combination of lifestyle management and regulating blood glucose levels and blood pressure, including drugs which inhibit or block the renin-angiotensin system. More recently, sodium-glucose cotransporter-2 (SGLT2) inhibitors have been established as a new class of glucose lowering agents which also reduce the incidence of DKD (Keri, Samji & Blumenthal, 2018). However, alternative options to prevent and treat DKD are still needed.

A variety of studies have demonstrated the beneficial effects on albuminuria when treating hyperglycaemia and hypertension (Ismail-Beigi et al., 2010). Hyperglycaemia, however, does not fully account for the development of DKD (Barnett, 2010). It is a combination of other factors including, hypertension, oxidative stress, proteinuria, dyslipidaemia, genetic predisposition, haemodynamic changes, duration of diabetes, obesity, weight, age, smoking and possibly gender. Over the last decade, there have been increasing amounts of research conducted to evaluate the factors contributing to the development and progression of DKD. Hence, it is timely for a contemporary review of the evidence available to be performed, which will have practice implications. This paper aims to review the factors which contribute to the development and progression of DKD in T2DM.

### Survey methodology

A literature search of EMBASE, ProQuest, PubMed, Science Direct and the Curtin University Library databases was undertaken using keyword searches (i.e., ‘type 2 diabetes mellitus’, ‘diabetic nephropathy’, ‘diabetic kidney disease’, ‘pathogenesis’, ‘risk factors’). Articles were restricted to those that were peer-reviewed, published in English with the full text available. Titles and abstracts were screened to determine relevancy.

## Discussion

The factors contributing to the development of DKD can be classified as modifiable and non-modifiable. Modifiable factors include hyperglycaemia and the mechanisms by which hyperglycaemia invokes functional and structural changes on the kidney, hypertension, dyslipidaemia, obesity, weight and smoking. Lifestyle modifications are crucial for controlling and minimising the impact of these factors, however when lifestyle management is insufficient, pharmacological strategies are used to provide a synergistic effect. Non-modifiable factors include age, duration of diabetes, gender and most importantly, the genetic make-up of the patient. Diabetes and its associated complications involve a variety of processes and body systems, hence are known as multi-factorial diseases.

**Figure 1:**
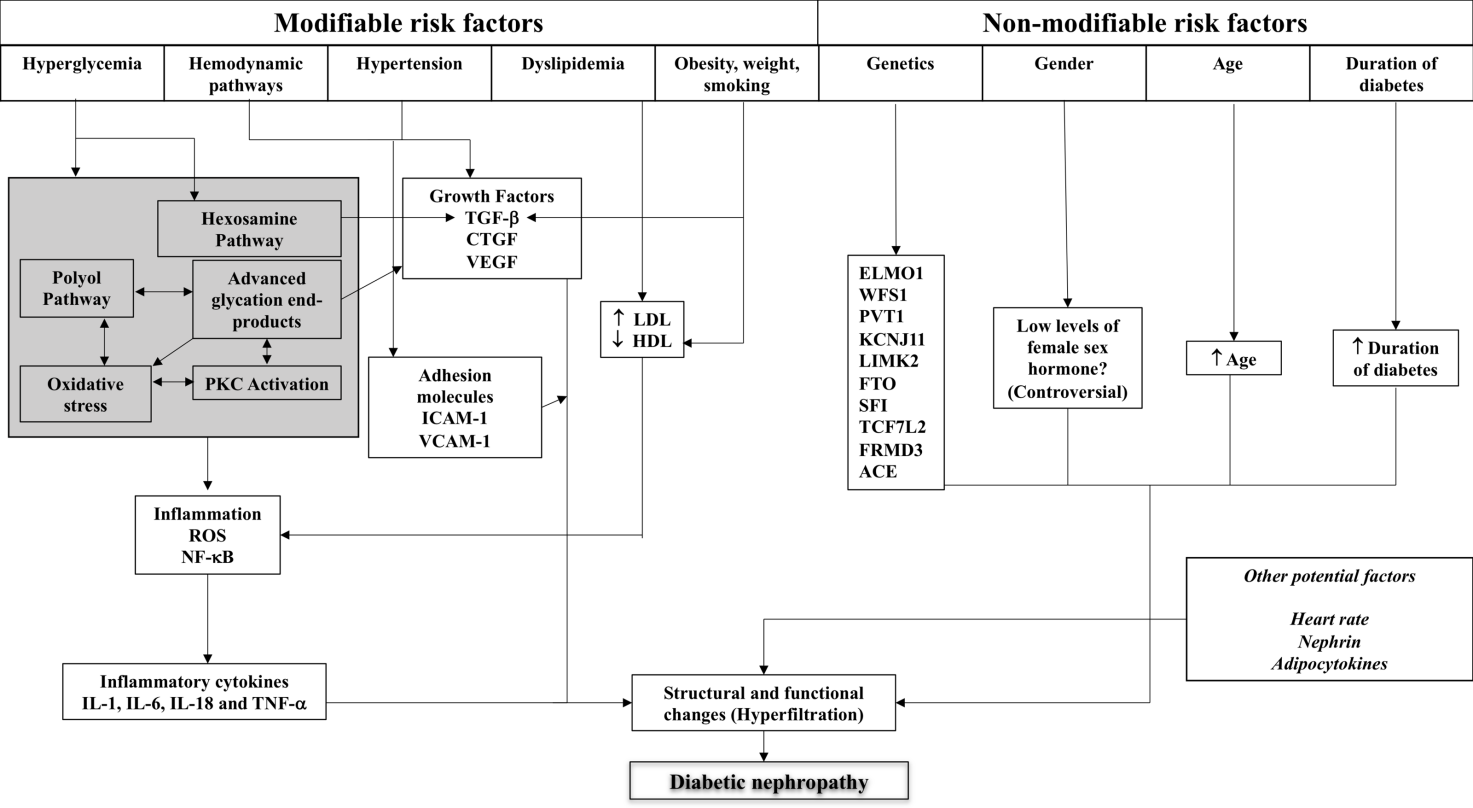
Schematic representation of the complex interplay of pathogenic mechanisms and patient-related factors in diabetic kidney disease. ACE, angiotensin-converting enzyme; CTGF, connective tissue growth factor; ELMO1, engulfment and cell motility 1; HDL, high density lipoprotein; ICAM-1, intercellular adhesion molecule-1; IL, interleukin; LDL, low density lipoprotein; NF-*κ*B, nuclear factor-kappa B; PKC, protein kinase C; RAS, renin-angiotensin system; ROS, reactive oxygen species; TGF, transforming growth factor; TNF, tumour necrosis factor; VEGF, vascular endothelial growth factor.

The details of these factors and their role in structural and functional changes in the kidney are described below, with Fig. 1 illustrating the complex interplay of these factors.

### Hyperglycaemia

Hyperglycaemia is a critical factor largely responsible in the pathogenesis of DKD. When intracellular glucose is not adequately dealt with through glycolysis, the activation of various pathways occurs, which has detrimental effects to the kidneys in particular the glomerular cells; parietal epithelial cells, podocytes, endothelial cells and mesangial cells (Song et al., 2019). These cells are responsible for glomerular capillary structure and modulation of glomerular filtration. Chronic hyperglycaemia is related to increases in mesangial cell proliferation and hypertrophy, matrix production and basement thickening (Song et al., 2019). In conjunction, fluctuations in blood glucose levels poses a risk for developing DKD (Yamagishi et al., 2007). The incidence of DKD in patients living with diabetes is significantly higher for those with glycosylated haemoglobin (HbA1c) levels above 7% or 53 mmol/mol (Fawwad et al., 2018). The pathways activated during hyperglycaemia include the generation of advanced glycation end products (AGE), the polyol pathway, hexosamine pathway and protein kinase C (PKC) activation and are discussed in detail below.

Anti-hyperglycaemic agents have a role in preventing the development of DKD however agents such as glucagon-like peptide-1 receptor (GLP-1R) agonists have been shown to prevent the onset of microalbuminuria independently of glycaemic effects (Rowlands et al., 2018). They work traditionally through glucose and blood pressure controls, reducing insulin levels and sensitivity and causing weight loss to delay the progression of DKD. More importantly, they have been shown to modulate inflammation in the kidneys and blood vessels, reduce renal oxidative stress, therefore reducing microalbuminuria (Thomas, 2017). Current evidence shows GLP-1R agonists exerting beneficial renal hemodynamic effects, however further investigations are required to understand the mechanism behind this (Rowlands et al., 2018; Thomas, 2017).

### Advanced glycation end products (AGEs)

During hyperglycaemia, excess glucose combines with free amino acids on circulating tissue proteins. AGEs are generated from this process. This is a non-enzymatic process ultimately affecting the glomerular basement membrane and other matrix components in the glomerulus (Keri, Samji & Blumenthal, 2018). Through this pathway, it is assumed that AGEs have the potential to alter the structure and function of the kidneys and initiate morphological changes typical of DKD (Yamagishi et al., 2007). Levels of AGEs are considerably higher in T2DM patients compared to those without diabetes (Keri, Samji & Blumenthal, 2018). Furthermore, patients with ESKD had almost double the amount of AGEs than those without renal disease, possibly due to impaired clearance of AGEs in renal failure. This suggests a possible correlation between concentrations of AGEs and the severity of DKD (Keri, Samji & Blumenthal, 2018). It has been postulated that AGE accumulation in the kidney may be responsible for the progressive decline in renal function (Bohlender et al., 2005). A number of AGE-binding proteins have been identified including galectin-3 (Vlassara et al., 1995), known as AGE-R3, OST-48 (AGE-R1), 80K-H (AGE-R2) (Li et al., 1996), class A and B macrophage scavenger receptors (Horiuchi et al., 1996; Ohgami et al., 2001) and receptor for AGE (RAGE) (Stern et al., 2002).

Receptor interaction with AGEs are also involved in the development of DKD. RAGE is a signal transduction receptor that mediates inflammatory reactions caused by AGEs. Podocytes and mesangial cells in patients with DKD have increased expression of RAGE (Yuan, Sun & Sun, 2017). In DKD, AGEs bind to RAGE, causing nicotinamide adenine dinucleotide phosphate (NADPH) oxidase to produce reactive oxygen species (ROS). Other signalling molecules are then activated, such as PKC, mitogen-activated protein kinase (MAPK) and nuclear factor-kappa B (NF-*κ*B). This eventually leads to increased expression of growth factors and cytokines, specifically transforming growth factor-*β* (TGF-*β*), vascular endothelial growth factor (VEGF), connective tissue growth factor (CTGF), platelet-derived growth factor (PDGF) and monocyte chemoattractant protein-1 (MCP-1), leading to AGEs-induced renal damage (Fukami et al., 2008). In conjunction, changes in the human kidney such as glomerular hypertrophy, glomerular basement membrane thickening, expansion of the mesangial matrix and overexpression of CTGF and NF-*κ*B activation were seen in diabetes-induced mice. These changes were blocked by the administration of a neutralising antibody to RAGE (Jensen et al., 2006). The AGE-RAGE interaction induces oxidative stress, causing vascular inflammation and playing a role in DKD. Oxidative stress increases concentrations of ROS, which encourages inflammation and fibrosis in kidneys. Increased expression of ROS also increases the synthesis of TGF-*β* and CTGF occurring in the mesangial and renal tubulointerstitial cells (Qin et al., 2019).

### Polyol pathway

In this pathway, aldose reductase produces sorbitol from glucose and sorbitol dehydrogenase oxidises sorbitol into fructose. These enzymes require NADPH and nicotinamide adenine dinucleotide (NAD+) for the successful conversion of glucose to fructose. The activity of the polyol pathway increases during states of hyperglycaemia, which decreases NADPH levels that are responsible for the regeneration of the antioxidant, glutathione (GSH) (Yan, 2018). In conjunction, imbalances exist between the glycogenesis and glycolysis pathways when high glucose concentrations are present. This results in a build-up of sorbitol, however sorbitol cannot easily penetrate the cellular membrane. This becomes even more difficult to process further once sorbitol is oxidised to fructose. Therefore, fructose and sorbitol accumulate in cells, altering renal osmolarity and potentially causing glomerular, tubular and tubulointerstitial injury. Tubulointerstitial injury also results from uric acid, which is generated from a side-chain reaction from fructose metabolism. In animal models, it has been demonstrated that blocking fructose metabolism reduced tubulointerstitial injury by lowering tubular uric acid production (Bjornstad et al., 2015; Nakagawa et al., 2020).

ROS production in glomerular and tubular cells has the potential to inflict free radical stress. Free radicals are removed via glutathione, which is reduced via this pathway during states of hyperglycaemia. Free radicals and ROS accumulate, disturbances in antioxidants occur exerting damaging effects on kidneys (Yan, 2018). Aldose reductase is the catalyst that triggers the polyol pathway. Inhibition of aldose reductase is a promising novel discovery that poses significant therapeutic opportunities for the management of DKD. This can be achieved by aldose reductase inhibitors, as they reduce sorbitol flux through the polyol pathway. A recent study investigated the effects of epalrestat on renal blood flow parameters in early DKD and found improvements in renal arterial blood flow and perfusion, which play a protective role in DKD (Liu et al., 2020).

### Hexosamine pathway

The majority of glucose is metabolised through glycolysis with only 2–5% of glucose entering the hexosamine pathway. When this pathway is activated, glucose enters the cell and is metabolised by glycolysis, converting fructose-6-phosphate to glucosamine-6-phosphate (GlucN-6-P) via glutamine: fructose-6-phosphate-amidotransferase (GFAT). This is the rate-limiting step. GlucN-6-P undergoes further processing to uridine-5-diphosphate-N-acetylglucosamine (UDP-GlucNAc). UDP-GlucNAc is the precursor for all amino sugars involved in the production of glycoproteins, glycolipids, proteoglycans and glycosaminoglycans. Increased flux of glucose through this pathway has been demonstrated to be involved in insulin resistance and the production of cytokines, growth factors and ROS (Schleicher & Weigert, 2000). In cultured mesangial cells that are exposed to high glucose levels, production of TGF-*β*1 is increased, leading to renal matrix expansion and renal scarring. Not only does TGF-*β*1 stimulate matrix synthesis, it is also responsible for impaired matrix degradation and therefore involved in tubulointerstital injury and glomerulosclerosis in T2DM. To confirm the involvement of the hexosamine pathway in high-glucose induced TGF-*β*1 synthesis, GFAT activity was blocked in mesangial cells (Schleicher & Weigert, 2000). This resulted in reduced levels of glucosamine metabolites and TGF-*β*1 mRNA and ameliorated their subsequent effects on the matrix. GFAT inhibition did not eliminate glucosamine-induced events however, due to the fact that glucosamine (GlucN) can bypass the rate-limiting step. Instead, GlucN can be phosphorylated by hexokinase to produce GlucN-6-P (Schleicher & Weigert, 2000). GFAT has also been overexpressed in NIH-3T3 fibroblasts to further elucidate the involvement of this pathway in TGF-*β*1 production and subsequent renal damage (Schleicher & Weigert, 2000). Upon overexpression of GFAT, cellular concentrations of UDP-GlucNAc were increased, indicating an increased flux through this pathway. In conjunction, TGF-*β*1 mRNA was also increased (Schleicher & Weigert, 2000). The hexosamine pathway has a role in DKD through its effect on TGF-*β*1 synthesis, however this role could be considered minimal compared to the effects of AGEs and the polyol pathway above, the other factors below and the fact that only 2–5% of all glucose enters this pathway (Schleicher & Weigert, 2000).

### Protein kinase C activation

Hyperglycaemia can lead to the activation of PKC through the formation of diacylglycerol (DAG) and oxidative stress. This pathway is responsible for the regulation of permeability, extracellular matrix (ECM), cell growth, cytokine stimulation, leukocyte adhesion and angiogenesis, which are all atypical in T2DM (King & Das-Evcimen, 2010). It has been proposed that PKC activation may enhance angiotensin II and vasodilatory prostaglandin synthesis. Both increase glomerular filtration rate (GFR) and filtration pressure, therefore increased activity may result in persistently high glomerular filtration pressure; predisposing to development of DKD. Extracellular matrix (ECM) accumulation and thickening of the basement membrane is a hallmark of DKD. PKC expression induces overexpression of fibrotic growth factors, in particular TGF-*β*1, which plays a role in ECM accumulation. Studies have demonstrated a linear relationship between PKC activation and TGF-*β*1 expression, leading to ECM production and the synthesis of type IV collagen and fibronectin (Yamagishi et al., 2007). In conjunction, capillary permeability is also altered due to PKC activation. PKC*β*1, a PKC isoform, has also been found to increase the permeability to albumin and other macromolecules in endothelial cells, ultimately altering kidney function (King & Das-Evcimen, 2010). Thus, the use of PKC*β* inhibitors may be an innovative strategy to improve outcomes in people living with T2DM and DKD. Experimental models have shown ruboxistaurin, a PKC*β* inhibitor, preserves kidney function by reducing urinary albumin excretion, reducing mesangial expansion and glomerulosclerosis and normalising tubulointerstitial fibrosis (Ishii et al., 1996; Kelly et al., 2007). These effects have been observed with ruboxistaurin alone, and in combination with renin-angiotensin system (RAS) inhibition. The findings from animal models have also been seen in small-scale clinical trials. Reductions in urinary albumin and stabilization of estimated glomerular filtration rate (eGFR) and TGF-*β* was seen after the addition of ruboxistaurin to angiotensin-converting enzyme (ACE) inhibitor (ACEi) and/or angiotensin receptor blocker (or antagonist, ARB) therapy in people with T2DM and DKD (Gilbert et al., 2007; Tuttle et al., 2005).

### Oxidative stress

During periods of high glucose concentrations, the polyol pathway, AGEs, PKC and hexosamine pathway become major sources of ROS. The relationship between ROS and DKD has been established and are significant players in the pathogenesis due to their ability to directly oxidise and damage DNA, carbohydrates, lipids and proteins. Mesangial, tubular, endothelial and vascular cells are all capable of synthesising ROS during hyperglycaemia however, dysfunction of vascular endothelial cells is seen in early DKD (Miranda-Diaz et al., 2016). Damage to podocytes and apoptosis has been identified from hyperglycaemia-induced ROS synthesis. Podocytes have been found to have a role in proteinuria and the number of podocytes is reduced in DKD. Structural changes also occur due to ROS including glomerular basement membrane thickening, mesangial expansion ultimately resulting in glomerulosclerosis (Sifuentes-Franco et al., 2018). ROS are usually counterbalanced by antioxidant enzymes (glutathione peroxidase, superoxide dismutase and catalase) and free radical scavenging systems. During hyperglycaemia, this balance is tipped in favour of ROS production (Miranda-Diaz et al., 2016). Furthermore, kidney biopsy specimens found more products of glyco-oxidation and lipoxidation in the glomerulus and mesangial matrix in patients with diabetes than those without. These lesions were far less common in non-diabetic specimens, indicating high-glucose environments increase the occurrence of oxidative stress and therefore kidney damage (Vasavada & Agarwal, 2005).

A number of studies have investigated the antioxidant defence mechanisms and shown that they may be potential targets for therapy. Antioxidant mechanisms include Keap-Nrf2 (nuclear factor erythroid 2-related factor 2), glutathione peroxidase, superoxide dismutase, catalase, co-enzyme Q and dietary antioxidants. Clinical trials have investigated Nrf2 activators and Lipoxin A4 to target Keap-Nrf2 (Chan, Han & Kan, 2001; Yang, Zheng & Hou, 2019) and glutathione peroxidase-1 (GPx1) mimics to target glutathione peroxidase (De Haan et al., 2003). Other clinical trials have investigated coenzyme Q10 (Sun et al., 2019), vitamins C (Klein, Juhl & Christiansen, 1995; McAuliffe et al., 1998) and E (Haghighat et al., 2014; Lonn et al., 2002), zinc (Khan et al., 2013) and magnesium (Farvid et al., 2005) as antioxidant supplementation in DKD. There appears to be benefits from oral antioxidant therapy in the reduction of ROS and oxidative stress however, it remains ambiguous as to which combination and dosage is the most effective.

### Hypertension

Blood pressure (BP) control is currently part of the first-line treatment for managing DKD in T2DM. High blood pressure has significant consequences on the integrity of the endothelium and plays a role in the development of DKD. Hypertension may be directly responsible for glomerular sclerosis, due to the increase in intraglomerular pressure. This leads to proliferation of mesangial cells and then eventually to the thickening of the basal membrane (Magee et al., 2017). Angiotensin II and endothelin, both peptide hormones are responsible for vasoconstriction and increased blood pressure. Endothelin-1 (ET-1) also functions to maintain and regulate renal water and salt homeostasis, however excessive production of ET-1 promotes proteinuria and tubulointerstitial injury, contributing to the development of albuminuria. ET-1 also causes fibrosis, inflammation, nephrin loss, podocyte injury and oxidative stress (Barton & Sorokin, 2015; Inscho et al., 2005). Endothelin-receptor antagonists, such as atrasentan, are potentially new therapeutic options in the management of DKD as they inhibit Janus kinases (JAKs) and signal transducers and activators of transcription (STATS) or otherwise known as the JAK/STAT pathway, MAP kinase, Nuclear factor-*κ*B (NF-*κ*B) and other intracellular signalling (Andress et al., 2012; Egido et al., 2017; Kohan & Pollock, 2013).

ACE converts angiotensin I (Ang I) to angiotensin II (Ang II) and ACE2 converts Ang II to angiotensin 1-7 (Ang1-7). Ang I and Ang II exert adverse effects in the kidneys through binding to the type 1 angiotensin receptor (AT1R); binding of Ang II to AT1R is a key driving factor in the development of DKD (Batlle et al., 2012). The effects of Ang II can be counteracted by signalling through the angiotensin II type 2 receptor (AT2R). It forms the “protective branch” of the RAS together with ACE2/Ang 1-7/mas receptor axis (Batlle et al., 2012; Ferrario & Varagic, 2010). Ang II is responsible for the adrenal gland releasing aldosterone, and aldosterone may further increase the risk of DKD through activation of mineralocorticoid receptors (MR) (Rossi et al., 2006). In addition, it has been found that ACE2 expression is decreased in proximal tubules, with ACE2 deficiency resulting in worsening of DKD. Notably, overexpression of ACE2 attenuates DKD (Soler et al., 2007; Tikellis et al., 2008). ACE, ACE2, Ang II and Ang 1-7 contribute to the development of DKD and could therefore potentially become targets in the management of DKD.

As mentioned previously, aldosterone and MR may also play key roles in the pathophysiology of DKD. MR are found throughout the body, but are strongly expressed in the kidneys, specifically in endothelial cells, glomerular mesangial cells, proximal tubular cells and podocytes (Nishimoto & Fujita, 2015; Nishiyama et al., 2005; Shibata, Ishizawa & Uchida, 2017). Aldosterone functions to maintain body fluid homeostasis however it may be a target for improving outcomes in people with DKD (Rossi et al., 2006; Sechi et al., 2006). MR activation causes a number of systemic changes such as hypertension, insulin resistance and local renal injury. Aldosterone-induced cell injury has been prevented through MR inhibition (Narayan & Webb, 2016; Sato, Saruta & Funder, 2006). RAS inhibition is a method for reducing inappropriately high plasma aldosterone levels however long-term treatment results in “aldosterone breakthrough” in some patients (Sato & Saruta, 2003). This phenomenon involves plasma aldosterone levels returning to pre-treatment levels. Aldosterone breakthrough attenuates the reno-protective effects of RAS inhibitors, leading to renal injury. The addition of MR antagonists or aldosterone antagonists to current therapy may be a novel treatment for DKD. The addition of MR antagonists has reduced albuminuria in people with T2DM and kidney disease and people with non-diabetic chronic kidney disease (CKD) (Mehdi et al., 2009; Vukadinovic et al., 2017). Unfortunately, prescribing either spironolactone or eplerenone should be done with caution and close monitoring is required in patients with CKD, due to the high risk of developing hyperkalaemia. Non-steroidal MR antagonists are currently being evaluated for use in DKD (Eschalier et al., 2013).

Although a full discussion of the renoprotective benefits of various therapies is outside the scope of this paper, administration with an ACEi or ARB has been proven to preserve kidney function (Blomster et al., 2016); this implies glomerular hypertension and hyperfiltration playing a role in the development of DKD. The effects of angiotensin II are antagonised by blockade of the renin-angiotensin-aldosterone system, therefore reducing its stimulation on TGF-*β*1. It has also been found that administration of an ACE inhibitor lowered serum concentrations of TGF-*β*1. A lower TGF-*β*1 concentration in the serum and urine has been correlated to renoprotection, which has been determined by changes in the glomerular filtration rate over time (Dronavalli, Duka & Bakris, 2008).

Previous studies have identified that a systolic BP (SBP) >140 mmHg significantly increases the risk of poor kidney outcomes. The risk was considerably greater in SBP of 151–160 mmHg than those of 131–140 mmHg (Pohl et al., 2005) and current American Diabetes Association guidelines recommend SBP <140 mmHg and diastolic BP (DBP) <90mmHg in most people living with diabetes. For those at high risk of cardiovascular disease, targets of 130/80mmHG may be more appropriate (American Diabetes, 2018). Recent studies have demonstrated the beneficial effect a lower BP on the kidneys in people with diabetes. For every 10 mmHg increase in the average systolic BP, the risk for further decline in the eGFR, developing ESKD or death, increased by 22%. Findings regarding the benefits of reductions in DBP are conflicting (Alwakeel et al., 2011; Leehey et al., 2015). This may be due to T2DM patients suffering with isolated systolic hypertension. While the use of ACEis and ARBs in delaying the progression of DKD in hypertensive T2DM patients is well established, the use of these agents in normotensive patients remains controversial. A recent meta-analysis investigated the efficacy and safety of these therapeutic agents in normotensive patients with DKD and found they significantly reduced albuminuria independent of changes to blood pressure (He et al., 2020). There were no significant differences in adverse effect profiles of the treatments (hyperkalaemia and hypotension) and response to treatment was superior in normotensive people living with T2DM than in normotensive patients with DKD (He et al., 2020). Conclusively, BP management does affect the development and progression of DKD and effective management is crucial in improving prognosis (McGrath & Edi, 2019).

### Dyslipidaemia and lipid mediators

The conversion of fatty acids to triglycerides in the liver is stimulated by insulin. It is therefore expected that insulin resistance would play a profound effect on lipid levels, profiles and fatty-acid composition. Dyslipidaemia has been associated with a greater risk for the development of DKD. This is supported through research that found the risk for DKD to be greater in patients with high levels of cholesterol, low-density lipoproteins (LDL) and triglycerides (Srivastava et al., 2014). Characteristics of dyslipidaemia in diabetes involve elevations of remnant lipoproteins, including very low-density lipoprotein (VLDL) and intermediate density lipoprotein (IDL), and a number of therapies are available to manage dyslipidaemia in people living with T2DM and DKD (Jenkins et al., 2003). Fenofibrate has been found to effectively reduce urine albumin excretion (UAE) and lower the decline in eGFR in people living with T2DM (Davis et al., 2011). A trial conducted by Elajami et al. explored the effects of eicosapentaenoic and docosahexaenoic acids (EPA and DHA, respectively) on albuminuria in patients with T2DM. Supplementation of 1.86 grams of EPA and 1.5 grams of DHA decreased the progression of albuminuria in people with T2DM, with the majority of patients also on RAS inhibition (Elajami et al., 2017). Supplementation with EPA and DHA may be beneficial in the management of DKD, however further investigations are necessary.

There is conflicting evidence surrounding the use of statins to treat dyslipidaemia in DKD, with some studies showing a benefit (Colhoun et al., 2009) and others not (Baigent et al., 2011), however people living with T2DM are at a higher risk for cardiovascular mortality. There is sufficient evidence illustrating the benefits of statins in reducing the risk of cardiovascular events (Baigent et al., 2011) therefore guidelines recommend the routine use of statins in people with T2DM and high cardiovascular risk, regardless of the presence or absence of DKD.

Animal studies have investigated the effects of small lipids derived from arachidonic acid in the pathogenesis of DKD. Arachidonic acid is metabolised into prostanoids via cyclo-oxygenase 2 (COX-2). Diabetes-induced rats were found to have increased levels of prostaglandins E_2_ and I_2_ (Komers et al., 2005) and COX-2 expression was found to be increased in diabetic animals and in diabetic human kidneys (Khan et al., 2001). In conjunction, lipoxygenases oxidise arachidonic acid and there is some evidence suggesting that these end-products may contribute to the development of DKD. Concentrations of lipoxygenase 12 and 15 was increased in diabetic animals and their expression in cultured mesangial cells was also elevated in the presence of high glucose levels. Furthermore, these pathways facilitate mesangial cell hypertrophy, which is mediated by TGF-*β*1 and angiotensin II (Hao & Breyer, 2007). Further investigations on the effects of prostanoids and lipid mediators in the development of DKD is needed before further conclusions can be made.

### Hemodynamic pathways and cytokines

Stimulation of the renin-angiotensin-aldosterone and endothelin systems, increases the secretion of TGF-*β*1 and other cytokines, ultimately resulting in increased systemic and intraglomerular pressure (Navarro-Gonzalez et al., 2011). The afferent and efferent arterioles in the glomerulus are affected first in glomerular hyperperfusion and hyperfiltration. Hyperfiltration results from the complex interplay of numerous mechanisms and mediators, with a prominent role for hyperglycaemia, distorted insulin levels and angiogenesis (Cachat et al., 2015). These play a role in part in the pathogenesis of hyperfiltration-induced renal damage (Cachat et al., 2015). There is a decreased resistance which may be due to prostanoids, nitric oxide (NO), angiotensin II, TGF-*β*1 and vascular endothelial growth factor (VEGF). These changes allow for albumin leakage, overproduction of mesangial cell matrix, glomerular basement thickening and podocyte injury. Renal hemodynamic changes may increase mechanical strain and induce the release of cytokines and growth factors in the local area (Ziyadeh & Wolf, 2008). Studies have suggested that activation of cytokines, inflammation and VEGF may be involved in the development of DKD, although results are inconclusive regarding the role of VEGF. VEGF expression is mediated by hyperglycaemia, TGF-*β*1 and angiotensin II, therefore leading to the production of endothelial NO. The result is vasodilation and hyperfiltration as seen in the beginning stages of DKD (Cao & Cooper, 2011). VEGF is also the primary and most potent mediator of abnormal angiogenesis in the glomerulus and may be a target for future therapies. VEGF-A is part of the VEGF family and is significantly involved in the physiological and pathological angiogenesis. Studies have found increased levels of VEGF-A in the urine and plasma of patients with DKD and a correlation between increased circulating VEGF-A levels and glycaemic control, C-reactive protein and albuminuria (Kim et al., 2005). This may mean VEGF-A has a role as a biomarker of inflammation and DKD in diabetes (Hanefeld et al., 2016). However, despite the promising results from anti-VEGF antibody studies conducted in animal models (DeVriese et al. 2001; Flyvbjerg et al., 2002), there is concern for its use in humans. Recent clinical findings have shown that the suppression of VEGF-A to subnormal levels results in proteinuria, hypertension and renal thrombotic microangiopathy (Feliu et al., 2015; Lafayette et al., 2014). Other potential anti-angiogenic therapies that may be favourable candidates for DKD treatment include endostatin and vasohibin-1 (VASH1). In the diabetic glomeruli, endostatin is likely to suppress the angiogenic response without the excessive VEGF-A inhibition that ultimately leads to proteinuria and renal dysfunction (Carlsson et al., 2016). Comparably, VASH1 exerts both antiangiogenic and endothelial protective effects, thus, has the potential to be a favourable candidate for antiangiogenic therapy (Watatani et al., 2014).

The expression of TGF-*β*1 is also increased with high blood glucose levels. In a study involving diabetes-induced rats, TGF-*β*1 levels in the glomeruli were increased. Administering a neutralizing antibody to TGF-*β*1 averted renal changes in these animals (Benigni et al., 2003). In conjunction, successful normalisation of urine protein levels of rats occurred. The combination of an ACE inhibitor plus an antibody to TGF-*β*1 was superior in normalising urine protein levels than the ACE inhibitor alone (Benigni et al., 2003). CTGF mediates the effects of TGF-*β* in conjunction with direct profibrotic activity. It has been found that mesangial matrix expansion was reduced in the diabetic murine models with down-regulation of CTGF expression (Guha et al., 2007). TGF- *β* signalling is inhibited through inhibitory Smads (I-Smads) through type I receptors, specifically Smad7 and by competing with receptor-regulated Smads (R-Smads) for receptor activation (Miyazawa & Miyazono, 2017).

The innate immune system has been found to be involved in the inflammation process through toll-like receptors (TLRs), nucleotide-binding oligomerisation domain-like receptors (NLRs) and inflammasome (Leemans et al., 2014). These are found in monocytes, macrophages and dendritic cells as well as endothelial cells and fibroblasts. Coincidently, most of these receptors are expressed in renal tissue. TLRs are responsible for increasing the expression of pro-inflammatory cytokines with the help of NLRP3 and NALP3 inflammasome, to convert interleukin 1 (IL-1) and IL-18 to their biologically active forms (Kurts et al., 2013; Leemans et al., 2014). IL-1, IL-6, IL-18 and tumour necrosis factor-*α* (TNF-*α*) have all been identified as inflammatory cytokines contributing to the development and progression of DKD. These inflammatory cytokines have been found in higher concentrations systemically and in the urine of patients, establishing a positive correlation with the progression of this complication (Liu et al., 2015b). They work via several different mechanisms to facilitate this complication. IL-1 affects mesangial cell prostaglandin synthesis by altering intraglomerular hemodynamics. It also changes the expression of chemotactic factors and adhesion molecules. IL-6 has been associated with the development of glomerular basement membrane thickening whereas IL-18 is responsible for the synthesis of other inflammatory cytokines (IL-1, interferon *γ* and TNF-*α*) and may also potentiate endothelial cell apoptosis. TNF-*α* is a cytotoxin, responsible for direct kidney damage. It also has a role in early hypertrophy and hyperfunction in DKD as well as affecting apoptosis and endothelial permeability (Liu et al., 2015b).

Adhesion molecules such as intercellular adhesion molecule-1 (ICAM-1) has been associated with the development of DKD. ICAM-1 levels in the kidneys are elevated in DKD and have been associated with renal damage. ICAM-1 is present in endothelial cell and leukocytes, and their expression is increased upon cytokine stimulation. It has been suggested that modulating ICAM-1 may be an innovative approach to DKD management (Luis-Rodriguez et al., 2012).

Another adhesion molecule, VCAM-1, has also been identified as a potential factor in the development of DKD. VCAM-1 is responsible for adhesion of lymphocytes, monocytes, eosinophils and basophils to vascular endothelium. It has been suggested that VCAM-1 is involved in the development of several process such as rheumatoid arthritis, atherosclerosis and DKD. It has been shown that VCAM-1 concentrations are increased in patients with DKD, however further investigations are required to fully understand its role (Luis-Rodriguez et al., 2012).

### Endothelial dysfunction

The development and progression of DKD is also driven by endothelial dysfunction and diminished nitric oxide (NO) bioavailability. Endothelial cells (ECs) are responsible for many physiological functions, including mediation of innate and adaptive immunity, cell proliferation, permeability and homeostasis (Calles-Escandon & Cipolla, 2001). Disruption to endothelial function may therefore promote the progression of DKD. It has been found that endothelial dysfunction is exacerbated by obesity, hyperglycaemia, insulin resistance, microalbuminuria, hypertension, dyslipidaemia and inflammation (Calles-Escandon & Cipolla, 2001). Chemical mediators such as NO and endothelial nitric oxide synthase (eNOS) also play an important role in endothelial dysfunction, with increasing evidence of eNOS and NO dysfunction in DKD (Cheng et al., 2012; Komers & Anderson, 2003; Zhou et al., 2019). Hyperglycaemia impairs NO production and bioavailability in renal ECs, resulting in increased oxidative stress, promoting endothelial dysfunction and injury (Alpert et al., 2005). Consequently, the injured ECs may act as active signal transducers of inflammatory (IL-1, TNF-*α*), metabolic and hemodynamic factors (TGF-*β*) that modify the morphology and function of the vessel walls, potentially activating proliferative and inflammatory responses in DKD (Monteiro et al., 2020; Nakagawa et al., 2004). There are studies evaluating the potential for regenerative medicine as a strategy for EC regenerative therapy in DKD, however further research is required to determine the efficacy and safety in humans (Bassi et al., 2012; Ikarashi et al., 2005).

### Mitochondrial dysfunction

Second to the heart, the kidney is considered to contain the greatest density of mitochondria per mass of tissue so that it can efficiently function and maintain homeostasis of electrolytes, water and other compounds (Parikh et al., 2015; Rosca et al., 2008). The proximal tubule cells and thick ascending limb (TAL) cells of the loop of Henle require large amounts of adenosine triphosphate (ATP) to retain water, sodium and electrolytes and for pumping sodium, potassium and chloride into the renal interstitium, respectfully. Hence, kidney function deteriorates if there is loss of mitochondrial function (Parikh et al., 2015). Since glucose oxidation involves the mitochondrial tricarboxylic acid cycle (TCA) cycle and oxidative phosphorylation, mitochondrial function may be the key to understanding T2DM and its associated complications (Sharma, 2017). People with T2DM and DKD have been shown to have reductions in mitochondrial DNA (mtDNA) content in urine exosomes, which are obtained from podocytes and tubular epithelial cells. It has been suggested that reductions in urine exosome mtDNA may reflect reductions in renal epithelial mitochondrial content (Sharma et al., 2013).

Mitochondrial function is influenced by the 5′-adenosine monophosphate (AMP)-activated protein kinase (AMPK) pathway as it is responsible for maintaining cellular energy homeostasis. Activation of AMPK improves glucose entry into cells, reduces protein synthesis and stimulates PGC1a and mitochondrial biogenesis, therefore improving energy efficiency (Jeon, 2016). AMPK is regulated by the AMP/ATP ratio and is activated under low levels of ATP. Examinations of the diabetic kidney have shown a correlation between elevated ATP/AMP ratio and reduced AMPK. Notably, type 2 DKD and diet-induced obesity models found AMPK reductions. AMPK activation has been shown to have beneficial effects on kidney outcomes, specifically reducing albuminuria as well as reducing NADPH oxidase, an enzyme responsible for the production of ROS in the kidney. A recent study found CoQ10 had beneficial effects on mtROS in animal models (Dugan et al., 2013).

### Podocytes

Podocytes line the external surface of the glomerular basement membrane and form a meshwork known as a slit diaphragm (SD), which provides a barrier to prevent urinary protein loss. Podocytes are also involved in forming the filtration barrier, therefore podocyte injury, which leads to loss of adhesion, may be involved in the pathogenesis of DKD (Greka & Mundel, 2012; Mundel & Kriz, 1995; Mundel & Shankland, 2002). These processes may be catalysed by hyperglycaemia-induced production of ROS, which is responsible for podocyte apoptosis, glomerular hypertrophy and subsequent sclerosis. In conjunction, podocyte loss is shown to be interrelated with eNOS deficiency (Susztak et al., 2006). Branching from this, poorly controlled T2DM causes increased concentrations of growth hormone (GH), which are implicated in kidney hypertrophy and proteinuria during early DKD (Landau et al., 2003). A number of studies have demonstrated that loss of podocytes and podocyte injury has significant effects on renal outcomes. A reduction in podocytes of up to 20% r caused proliferation of the mesangial cells. Further reductions resulted in glomerular fibrosis and increased proteinuria (Wharram et al., 2005). Notably, it was found that podocyte density correlates to albuminuria (Dalla Vestra et al., 2003). Podocytes contain foot processes (FPs) that wrap around capillaries and leaves slits for filtration. Results from renal biopsies revealed widening of FPs coupled with reduced podocytes. Furthermore, podocytes have been found in the urine of patients with glomerulopathies and the magnitude of podocyte excretion correlates to DKD activity (Merscher & Fornoni, 2014; Yamaguchi et al., 2009).

One of the canonical Wnt signalling pathways, Wnt/*β*-catenin signalling, plays a role in podocyte dysfunction and proteinuria and is activated in a number of proteinuria kidney diseases (Liu et al., 2015a; Yang et al., 2015). A number of studies have shown activation of Wnt/*β*-catenin causes fibrosis in the kidney (Buelli et al., 2014; Liu et al., 2016; Lymperopoulos, 2012). Increases in *β*-catenin, a transcriptional regulator essential for kidney development, correlates to activation of Wnt/*β*-catenin signalling (Zhang et al., 2016). *β*-arrestins are involved in inhibiting G protein-coupled receptors and there is evidence that they participate in the development of DKD (Venkatakrishnan et al., 2016). A study found that *β*-arrestins were upregulated in podocytes subjected to high glucose and that *β*-arrestin 1/2 promoted podocyte apoptosis. In conjunction with this, *β*-catenin expression was increased in the *β*-arrestin 1/2 up-regulated group (Wang, Li & Song, 2018). It should be noted that inhibition of the Wnt/*β*-catenin pathway ameliorated apoptosis. From this study we can conclude that *β*-arrestin 1/2 has a role in podocyte apoptosis through the Wnt/*β*-catenin signalling pathway (Wang, Li & Song, 2018).

### Genetic susceptibility

The incidence and severity of DKD appears to be affected by the genetic makeup of individuals (Skrunes et al., 2014). Both environmental and genetic factors play a critical role in the development of diabetes, so these factors may also play a role in diabetes-related microvascular complications. The complexity of the genome involvement in DKD makes a complete discussion difficult within the scope of this paper. In summary, a combination of candidate gene investigations, linkage studies and genome-wide association studies (GWAS) has allowed us to understand the mediating role of the genome in DKD. DKD and ESKD cluster in families and the prevalence varies between ethnic groups (Skrunes et al., 2014). A single nucleotide polymorphism (SNP) has been associated with DKD. On chromosome 7q21-q22 in the promoter region of the erythropoietin (EPO) gene, the functional SNP rs1617640 has been associated with ESKD in a number of diabetic populations. EPO was found to increase the rate of kidney damage and the T allele of rs1617640 has been described as a ‘risk factor’ for DKD (odds ratio, 1.5) (Nazir et al., 2014). The engulfment and cell motility 1 (ELMO1) gene, located on chromosome 7p14.1, has a functional role in phagocytosis and cellular motility. Overexpression of ELMO1 may result in the progression of glomerulosclerosis and occurred in cultured cells present in a high glucose environment. It has been associated in a number of populations with DKD (Nazir et al., 2014). EMLO1 overexpression increases the expression of TGF-*β*1, resulting in the ROS formation and therefore DKD (odds ratio, 2.7) (Hathaway et al., 2016).

A variety of studies have identified mutations to the ACE gene contributes to the development and progression of DKD. It has been found that insertion/deletion polymorphism of ACE is common among patients and that deletion of the rs179975 polymorphism of ACE gene was associated with DKD in people with diabetes. The dysfunctional ACE gene is responsible for increases in aldosterone, causing toxicity and fibrosis of blood vessels (Vikram Rao et al., 2019).

Another gene that may be implicated in the development of DKD is the FRMD3 gene. This gene is expressed in proximal renal tubular cells and human podocytes and may be involved in maintaining the integrity and function of the slit diaphragm (Mueller et al., 2006; Pezzolesi et al., 2013). It has been demonstrated that the rs1888747 SNP is associated with DKD and is an intergenic polymorphism residing near the promoter region of the FRMD3 gene (Palmer & Freedman, 2013). Changes in FRMD3 expression has been associated with DKD progression and the effect of these changes in present prior to advanced stages of DKD, such as in microalbuminuria (Martini et al., 2013). A number of other genes have been identified from T2DM studies and include WFS1 (odds ratio, 1.04), FTO (odds ratio, 1.11), TCF7L2 (odds ratio, 1.00) and KCNJ11 (odds ratio, 1.14) (Franceschini et al., 2012; Yang et al., 2012). A comprehensive discussion of these genes is beyond the scope of this paper.

### Other modifiable and non-modifiable risk factors

A modifiable risk factor that may play a role in the development of DKD is obesity and becoming overweight. A variety of studies have identified patients with T2DM that were either overweight or obese, were at a greater risk for the development of DKD and ESKD (Al-Rubeaan et al., 2014). There are two classes of obesity; generalised obesity measured through body mass index (BMI) and abdominal obesity, assessed using either waist circumference and/or waist-to-hip/height ratio (Nguyen et al., 2011). Obesity is an established risk factor for the development of T2DM and hypertension, which is a risk factor for DKD however it remains uncertain which contributes more to the risk of DKD due to their close inter-relationship (Nguyen et al., 2011; Vazquez et al., 2007). A meta-analysis found a higher BMI was independently associated with a greater chance of developing DKD, and that both generalised and abdominal obesity are risk factors in the pathophysiology of DKD, independent of their roles in hypertension and T2DM (Brown et al., 2000; Jabbar et al., 1997). This risk factor is preventable and it is recommended that lifestyle modifications are part of diabetes management. Smoking is another modifiable risk factor with some studies demonstrating a higher odds ratio and relative risk in developing DKD (Yang et al., 2011) than others (Al-Rubeaan et al., 2014), which may potentially be due to a lower smoking prevalence in those population groups.

It has been documented that the likelihood of developing DKD increases with the duration of diabetes (Al-Rubeaan et al., 2014). It has been found that people living with diabetes for more than 10 years have a significantly higher prevalence of DKD than those living with diabetes for only 5-10 years (Wu, Chen & Chen, 2014). Some studies have identified diabetes duration to be one of the most important risk factors for developing DKD, especially if the duration ≥15 years (Al-Rubeaan et al., 2014). This has been demonstrated by the United Kingdom Prospective Diabetes Study (UKPDS), whereby microalbuminuria or worsening DKD developed in approximately one quarter of patients after 10 years (Adler et al., 2003). There have been reports of a cumulative effect between diabetes duration and age on the prevalence of DKD. The prevalence is increased by approximately 5 times in patients suffering with diabetes for longer than 15 years. This establishes a correlation between diabetes duration and age (Al-Rubeaan et al., 2014). Advancing age comes with other issues as body processes are conducted less efficiently. It has been found that an increasing age is associated with a faster decline in GFR. Despite this, older patients had a lower cumulative incidence of renal failure. The high prevalence of ESKD may be explained by the higher prevalence of chronic kidney disease (CKD) in these aging groups (Eriksen & Ingebretsen, 2006).

Male gender appears to have a negative effect on renal outcomes and this has been a matter of debate (Eriksen & Ingebretsen, 2006). It has been reported that men have a greater decline in GFR than women and also a higher prevalence of DKD (Al-Rubeaan et al., 2014). Despite these findings, the topic remains controversial due to the number of studies reporting diabetic women with DKD progressing at a faster rate than men or similar rates of progression between both sexes. It has been found that the incidence and progression of renal disease in premenopausal non-diabetic women was considerably lower than non-diabetic age-matched men. This relationship was not apparent in postmenopausal women. This may be due to low levels of the female sex hormone however strong evidence is lacking and it is not a major consideration clinically (Maric & Sullivan, 2008).

### Other factors and clinical implications

New factors are being postulated to have roles in DKD, however firm evidence is lacking and further investigation is required. These factors are potentially important to advance understanding and possibly for development of new treatments for DKD. Some of these factors are discussed below.

#### Heart rate

Heart rate (HR) is an under-explored contributor to the pathogenesis of DKD. A higher resting HR has been associated with an increased prevalence and severity of microalbuminuria, irrespective of beta blocker treatment and irrespective of whether a patient history of atrial fibrillation is present (Bohm et al., 2009). The Atherosclerosis Risk in Communities (ARIC) Study identified an increased HR as a predictor of renal dysfunction. The study concluded resting heart rates in the upper quarter would increase the risk of developing ESKD by 2-fold (Brotman et al., 2010). Similarly, a Japanese health screening program found a higher HR in participants was associated with an increased risk of declining eGFR and of developing dipstick proteinuria (≥1) (Inoue et al., 2009). The mechanism by which HR might promote microalbuminuria is possibly due to an increased exposure of the glomerulus to arterial pressure waves. An increased HR has several direct cardiovascular consequences including unfavourable effects on endothelial function and proatherosclerotic activity, which are fundamental factors in the pathogenesis and progression of DKD (Hillis et al., 2012). However, the relationship is complicated by the fact that obesity, hypertension, reduced physical activity and a proatherosclerotic lipid profile are all associated with a higher HR, while also independently increasing the risk of developing DKD. These are targets for intervention to improve outcomes in patients living with T2DM (Palatini, 2009). The relationship between HR and microvascular complications such as DKD, may be mediated at least in part by sympathetic overactivity and can be a target for intervention to improve outcomes in patients living with diabetes (Hillis et al., 2012).

#### Nephrin

Nephrin is a protein essential for the integrity of the intact filtration barrier. It is found in podocytes, which preserve the dynamic functional barrier. It has been hypothesised that nephrin may have a role in DKD, due to the reduced renal nephrin expression in patients with DKD compared to those without diabetes. Nephrin excretion is also increased by 17–30% in people with diabetes compared to those without, irrespective of whether albuminuria is present. Excretion of nephrin may therefore be an early finding of podocyte injury, prior to the development of albuminuria (Fiseha, 2015). Furthermore, nephrin expression was maintained in people with T2DM treated with ACE inhibitors, compared to untreated controls (Wolf, Chen & Ziyadeh, 2005), although more research is required to confirm the clinical importance of this finding.

#### Adipocytokines

Adiponectin is a protein hormone involved in regulating glucose levels and fatty acid breakdown and circulates as trimeric, hexameric and higher order complexes in plasma (Ceddia et al., 2005). It has been inversely correlated with insulin resistance and BMI. Experimental studies have illustrated that insulin sensitivity is improved with adiponectin through glucose utilization in the liver and skeletal muscles. This enables glucose uptake and prevents gluconeogenesis in the liver (Ceddia et al., 2005). In conjunction, inflammatory changes mediated through TNF-*α* are suppressed when adiponectin levels are high. Local adiponectin accumulation has been shown to prevent glomerular and tubulointerstitial injury through mediating inflammation and oxidative stress, while gene variations have also been associated with plasma isoforms and the risk of DKD (Ohashi et al., 2007).

Leptin is a hormone produced by adipose cells and has a primary role in energy uptake and expenditure in addition to potential inflammatory effects. It also has a role in body fat, obesity and weight regulation, which are major risk factors for the development of T2DM. Leptin acts upon transmembrane receptors, known as OB-R, that are structurally similar to the cytokine receptors, leading to activation of the JAK/STAT pathway (Hegyi et al., 2004; Sahu, 2003). There are four receptor-associated JAKs, JAK1, 2, 3 and tyrosine kinase 2 (Tyk2), and seven STAT proteins (STAT 1, 2, 3, 4, 5a, 5b and 6) (Stark & Darnell Jr, 2012). The binding of leptin to its receptor initiates the phosphorylation of JAK2, and subsequently phosphorylation and dimerization of STAT 3. STAT 3 induces transcription of the gene encoding proopiomelanocortin (POMC), whilst inhibiting expression of Agouti-related peptide (*Ag* RP) and neuropeptide Y (NPY). Whilst POMC and AgRP/NPY peptides possess opposite functions, they are critical for promoting changes in satiety in the neuronal pathways of the hypothalamus. In conjunction, STAT 3 stimulates the expression of SOCS (suppressor of cytokine signalling proteins), a feedback inhibitor of the leptin signalling pathway (Myers Jr et al., 2010; Zhang, Dodd & Tiganis, 2015). It has been reported that leptin plasma concentrations are increased in T2DM patients with micro- or macro-albuminuria, as well as in obese individuals. Hyperleptinaemia and leptin resistance is responsible for reduced effects of leptin as a satiety hormone and are implicated in suppressed insulin secretion in *β*-cells (Muniyappa et al., 2014; Myers Jr et al., 2010). Increases in leptin stimulation has been shown to increase ROS production, producing a linear relationship between the two and also stimulates TGF-*β*1 production (Fruehwald-Schultes et al., 1999). In normal rat populations, infusion of leptin was found to promote glomerulosclerosis and proteinuria, however studies in humans is lacking (Fruehwald-Schultes et al., 1999).

### Mechanisms currently targeted therapeutically

Currently there are a few of these mechanisms that are targeted therapeutically for the management of DKD and include hyperglycaemia, hypertension and some modifiable risk factors. Hyperglycaemia is currently managed with an array of medications such as sulfonylureas, thiazolidinediones, dipeptidyl peptidase-4 inhibitors, GLP-1 analogues, metformin and SGLT2 inhibitors (Keri, Samji & Blumenthal, 2018). In conjunction, antihypertensive medications have a role in reducing the development and progression of DKD, specifically ACE inhibitors and ARBs (American Diabetes, 2018). Targeting modifiable risk factors such as weight, obesity and smoking has beneficial effects on the prognosis of DKD (Al-Rubeaan et al., 2014). There is the potential for other factors to be targeted therapeutically such as heart rate however further investigations are required. Selonsertib, an apoptosis signal-regulating kinase 1 (ASK1) inhibitor, is currently been used in phase II clinical trials for the management of DKD (Lin et al., 2015). ASK1 is associated with apoptosis of podocytes, mesangial cells and tubular epithelial cells. It is also involved with inflammation and fibrosis of the kidney; hence it is believed that inhibition of ASK1 may reduce the pathological process which contributes to kidney injury (Gao et al., 2014). Other potential mechanisms are currently under investigation, with some ongoing studies exploring the efficacy of pentoxifylline (NCT03625648), colchicine (NCT02442921) and magnesium supplementation (NCT03824379). Pentoxifylline, a methylxanthine phosphodiesterase inhibitor, may protect the kidneys from damage resulting from T2DM and other chronic diseases (Shan et al., 2012). The ongoing clinical trial investigating pentoxifylline (NCT03625648) will determine if pentoxifylline can prevent worsening of diabetic kidney disease. In addition to pain relief in acute gout, colchicine has also been used to treat and reverse albuminuria in familial Mediterranean fever nephropathy (auto-inflammatory) disease (Oner et al., 2003; Zemer et al., 1986). It has successfully reduced proteinuria and inflammation in experimental-diabetic animal models and has the potential to be part of the management strategy in DKD (Milner et al., 1987). A current trial (NCT02442921) will assess if colchicine reduces proteinuria, in conjunction to RAS inhibition and tight glycaemic control. Magnesium supplementation through the administration of magnesium citrate is also under investigation for the treatment and management of DKD (NCT03824379). This is in response to hypomagnesemia and worsening kidney outcomes. Serum magnesium levels were significantly inversely correlated to UACR (Xu et al., 2013). Magnesium supplementation may also improve insulin resistance index (Morais et al., 2017), lipid profile and kidney function (Sadeghian et al., 2020). There are other ongoing trials investigating new agents for DKD, and include investigations into fexofenadine (NCT04224428), niclosamide (NCT04317430) and CSL346 (VEGF-B antagonist monoclonal antibody, NCT04419467).

## Conclusions

The development and progression of DKD is a result of the complex interplay between environmental, lifestyle and genetic factors, that impact the structure and function of the kidneys, impair normal physiological processes and increase concentrations of damaging cytokines. This understanding of diabetes and DKD as a multifactorial disease has the potential to change the way we think and manage such complications. A greater understanding of the disease among clinicians and researchers permits innovative research into potential therapies that will produce significant clinical benefits to people living with diabetes and reduce its economic burden.

## Additional Information and Declarations

### Data Availability

The following information was supplied regarding data availability: This is a literature review summarising the findings from other studies. References to the studies with raw data are cited.
